# Deletions in (CCCT)_n_ repeat regions belonging to the human pRNA gene inhibit its expression

**DOI:** 10.1186/s40246-025-00830-w

**Published:** 2025-10-28

**Authors:** Nikola Chmúrčiaková, Adam Nógell, Evgeny Smirnov, Dušan Cmarko

**Affiliations:** 1 Laboratory of Cell Biology, Institute of Biology and Medical Genetics, First Faculty of Medicine, Charles University and General University Hospital in Prague, Prague, Czech Republic; 2 Department of Genetics and Microbiology, Faculty of Science, Charles University, Prague, Czech Republic

**Keywords:** rDNA, pRNA, Short tandem repeats, Transcription, Triplex

## Abstract

**Background:**

Ribosomal DNA (rDNA), the most abundantly expressed locus in the human genome, is represented by hundreds of units per cell. Each unit includes a 13 kb long region (47S rDNA) containing the genes of ribosomal particles, and a 30 kb long intergenic spacer (IGS). The 47S rDNA is transcribed with varying intensity in different units, some of which remain permanently silent. A key intermediator of this silencing is the promoter-associated RNA (pRNA) produced from a 2 kb long gene situated upstream of the rDNA transcription start site. Recent studies, including ours, suggest that the sequence variability, which normally occurs in mammalian cells, may account for the selective transcription of different rDNA units. The present work is based on the deep sequencing of a pRNA gene fragment and its RNA product and subsequent bioinformatic analysis.

**Results:**

We found that a certain SNV, which converts the CCC motif into CCT, as well as deletions which reduce the number of (CCCT) tandem repeats, were significantly more frequent in the DNA than in the respective transcripts.

**Conclusions:**

These findings allowed us to establish directly the inhibitory effect of DNA variants on the expression of pRNA and thus (indirectly) the promoting effect on the production of ribosomal RNA. Our results also suggest that (CCCT)_n_/(GGGA)_n_ DNA repeats and the respective (GGGA)_n_ RNA repeats may form triplex structures, facilitating the function of pRNA.

## Background

**Fig. 1:**
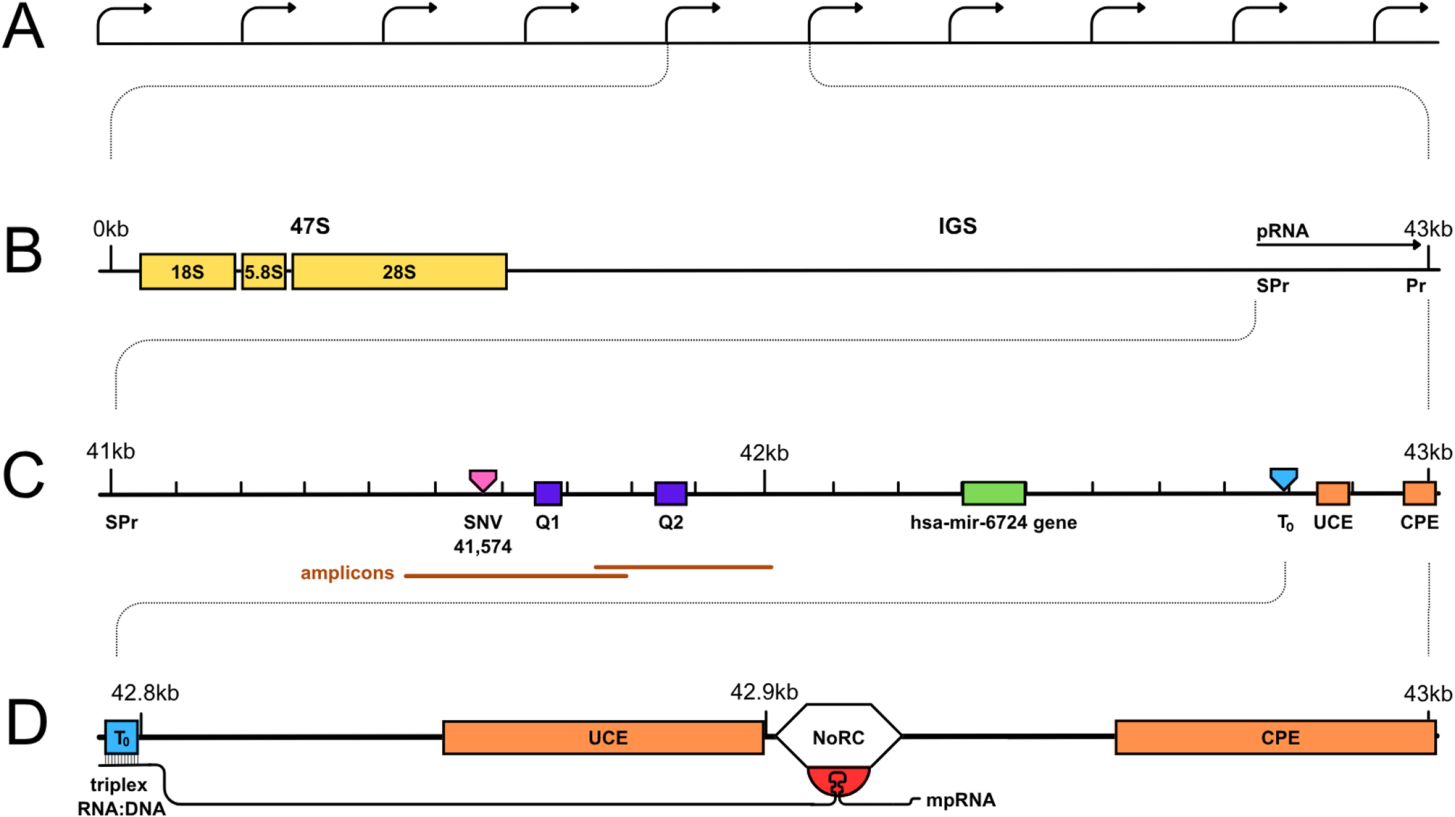
The structure of the human rDNA locus. **A** A cluster of the rDNA units. **B** Details of one unit including genes encoding 18S, 5.8S, and 28S ribosomal RNAs; intergenic spacer (IGS); rDNA promoter (Pr); spacer-promoter (SPr); the gene of promoter-associated RNA (pRNA). **C** The pRNA locus which includes the upstream control element (UCE) and the core promotor element (CPE) of the rDNA promoter; SNV_41,574_ (CCC > CCT) significantly correlating with DNA methylation status; regions of simple tandem repeats (CCCT)_n_ marked Q1 and Q2; promoter-proximal terminator (T_0_); the gene encoding hsa-mir-6724. **D** Interaction of the mature pRNA (mpRNA) with T_0_, by forming RNA:DNA triplex, and with the nucleolar remodelling complex (NoRC), by forming a specific hair-pin structure. In this conception, no role is assigned to the sequence lying between SPr and T_0_

Ribosomal DNA (rDNA), the most abundantly expressed locus in the human genome, is represented by hundreds of repeats or units per cell. Each unit consists of two parts: a 13 kb long 47S DNA containing the genes of ribosomal particles, and a 30 kb long intergenic spacer (IGS) (shown in Fig. 1A, B). The 47S DNA is transcribed by RNA polymerase I (pol I) with various intensity in different units, some of which remain permanently silent. The transcription is regulated, in the entire locus as well as in individual units, through multiple pathways targeting the state of pol I and its transcription factors or the chromatin structure of the promoter area [1–3].

The promoter-associated RNA (pRNA) is an important inhibitor of the rDNA transcription [4–6]. In murine and human cells, this long non-coding RNA is produced by pol I from the spacer promoter (SPr) located around 2 kb upstream of the transcription start site (TSS) [4] (shown in Fig. 1B, C). The coding region of pRNA starts at SPr and ends downstream of TSS. The primary 2,000 nt transcript is processed and reduced into a 150-250 nt mature pRNA (mpRNA) complementary to the region, which includes proximal terminator T_0_ as well as 47 S rDNA promoter with its upstream control element (UCE) and core promoter element (CPE) (shown in Fig. 1D). The mpRNA is stabilized by binding to TIP5 (transcription termination factor I interacting protein 5), the large subunit of the nucleolar remodelling complex (NoRC), which mediates transcriptional silencing via DNA methylation and histone modification [4, 7–10]. Apparently, two interactions are involved in the 47S rDNA silencing by the mpRNA. On the one hand, assuming a specific hairpin structure, mpRNA binds to TIP5 somewhere between UCE and CPE; on the other hand, the same RNA molecule recognizes the complementary double-strand DNA at T_0_, with which it forms a triplex. Some authors still question the existence of such triplex, indicating, among other things, that T_0_ sequence with its irregular alternation of purines and pyrimidines is not favourable for the triplex formation [11]. Nevertheless, associations of pRNA with T_0_, which also binds transcription termination factor I (TTF-1) and with NoRC, seem to be necessary for the subsequent recruitment of DNA methyltransferases and other factors introducing the repressive marks at the rDNA promoter area. The DNA:RNA triplex may stabilize the mpRNA and facilitate its specific interactions with NoRC and TTF-1 [4, 8, 12]. Expression of pRNA seems to be variously regulated as well. For example, TTF1 may arrest transcription from the SPr by acting as a “roadblock“ [13].

Results of recent studies indicate that the sequence variability, which normally occurs in mammalian cells, may account for the selective transcription of different 47S rDNA units [14]. Certain single nucleotide variants (SNVs), e.g. at the position -413 close to the promoter, correlate with the epigenetic marks and thus probably with the transcription activity [15–17]. Remarkably, very little attention has been paid so far to the upstream part of pRNA gene which does not belong to the mpRNA coding sequence. But this part, located between SPr and T_0_, contains a number of functional and potentially functional regions [14], including a whole micro-RNA gene, two sequences aligned with the exogenous micro-RNAs, two regions (Q1 and Q2) of short tandem repeats (CCCT)_n_ (shown in Fig. 1), apparently prone to quadruplex formation. In our earlier work, we found that deletions in Q1 and Q2, as well as certain SNVs in the pRNA gene, significantly correlate with DNA methylation, suggesting that these variants may be engaged in the regulation of rDNA transcription. In the present study, by comparing the sequence of a segment of the pRNA gene with the sequence of RNA produced from it, we established directly the effect of DNA variability on the expression of pRNA and thus (indirectly) on the production of ribosomal RNA. Our new data also compelled us to revise our views on the role of DNA methylation in the economy of the rDNA locus, and we accordingly corrected the hypothesis formulated in our previous work [14].

## Methods

### Cell culture

The human fibrosarcoma cell line, HT1080, was obtained from the American Type Culture Collection (Rockville, MD, USA). The human primary fibroblast cell line, WI38, was obtained from Merck (Darmstadt, Germany). The cells were maintained in complete DMEM Dulbecco’s modified Eagle medium containing 10% FBS and 1% penicillin-streptomycin. All the chemicals were obtained from Merck Life Science (Prague, Czech Republic) unless otherwise stated. Cell cultures were maintained at 37 °C in a humidified atmosphere of 5% CO_2_.

### Extraction of nucleic acids

Total DNA and RNA were sequentially extracted from the cells by TRIzol reagent (Invitrogen, Carlsbad, CA, USA) according to manufacturer’s instructions. Nucleic acids were quantified using the Eppendorf Biophotometer. Purity was considered sufficient if A260/A280 measured between 1.8 and 1.9 in DNA fractions and 1.9-2.0 in RNA fractions. RNA integration was additionally checked by gel electrophoresis, while 2 bands of 18S and 28S represented satisfactory RNA quality. Then cDNA was synthesised using a High-Capacity cDNA Reverse Transcription Kit (Thermo Fisher Scientific, Waltham, MA, USA).

### Amplification of regions Q1 and Q2 containing CCCT repeats

Both DNA and cDNA fractions were amplified by PCR with the primers listed in Table 1. PCR amplification was performed in 35 cycles, and the resulting amplicons were verified by agarose gel electrophoresis. The gels showed clean, strong bands at the expected positions, without visible degradation or nonspecific products.

**Table 1:** Primers used for PCR amplification and their position in reference to U13369.1

Position	Size of the product (bp)	Primers
41,469–41,790	321	F′ CGTTCCCTGTGTT TCCTTCT
		R′ GCAGAATCGGTAGGCTCTTC
41,748–42,033	285	F′ GTCTGTCTCTGC GTGGATTC
		R′ CGAAACCGTGAGTCGAGAAG

### Bioinformatic analysis

The amplicons were subjected to library preparation by the sequencing provider (Novogene Co., Ltd., Munich, Germany) who also performed the quality control. Standard Illumina-compatible library preparation protocols were used, including end repair, adapter ligation, and indexing. Libraries were subsequently pooled and sequenced on an Illumina NovaSeq6000 platform, using 250PE protocol. The sequencing output was of high quality across all samples, with Q30 scores above 70% and low error rates (0.05–0.09%). The sequencing data were processed by a combination of well-established tools and an in-house Python script. The sequencing adapters were removed using Cutadapt [18]. This step also included quality- and length-filtering – reads that were shorter than 60 bases were discarded as well as the bases that had a quality lower than 18. Reads of satisfactory quality were mapped to the reference rDNA sequence U13369.1 using BWA-MEM [19]. All reads that did not cover the Q1 or Q2 regions were filtered out using SAMtools [20]. Then clustering was performed by CD-HIT-EST [21, 22]. As the input files for the Python script, we used the clustered reads that were mapped again and transformed using their CIGAR strings to show better their deleted regions. The size of the reads was reduced so they would contain only the Q1 or Q2 regions with 5 bp long flanking sequences. The reads were finally clustered again, but this time with the set of Python functions instead of CD-HIT-EST, which better suited our case. The output of this script was a text file containing representative sequences of the clusters along with the numbers of their occurrence in the original file and percentage of the occurrence. In each sample, the output text file also contained a reference sequence cut at the same position as the examined reads.

## Results

1. Short variants in the entire CCCT-repeat-containing amplicons and the corresponding cDNA in HT1080 cells

After isolation of DNA and RNA from the cells, we used two pairs of primers to amplify a segment of pRNA gene, the sequence situated between the positions of 41,469 and 42,033 (referring to the human rDNA GenBank sequence U13369.1: https://www.ncbi.nlm.nih.gov/nuccore/U13369.1?report=graph&from=19514) containing two regions (Q1 and Q2) of CCCT repeats. The PCR products were subjected to deep sequencing to establish all variants and compare their frequencies in the DNA and the cDNA derived from the isolated RNA.

Examination of single nucleotide variants (SNVs) as well as short insertions and deletions (indels) in the regions of interest (shown in Fig. 1), revealed only one significant variant, C>T substitution at the position 41,574 (Fig. 1C; Table 2). This variant appeared with high frequency and reduced mononucleotide motif CCC to CCT. In our previous study [14] the same SNV_41,574_ showed a strong correlation with the DNA methylation status determined in the immunoprecipitation assay with antibody against 5-methylcytosine. In the present work we find that SNV_41,574_ occurs less frequently in the DNA than in the corresponding cDNA (Table 2).

**Table 2:** Occurrence of the C > T substitution (position 41,574) in DNA and cDNA

	Frequency	Coverage	Significance
DNA	0.68	6,590,000	*p* < 0.001
cDNA	0.95	1,830,000	
Meth*	0.48	1,195	*p* < 0.001
Non-meth*	0.69	4,467	

* Depicts frequency of reads with high level methylation (meth) and low level methylation (non-meth) DNA according to the data of Chmurciakova et al. 2022

(2) Variability of the CCCT repeat number in Q1 and Q2 regions and in the corresponding cDNA

To analyse the variability in the regions Q1 and Q2, we selected reads covering these microsatellite regions with at least 5 bp long flanking sequences. The observed variability was, for the most part, related to the number of CCCT repeats (shown in Table 3).

**Table 3:**
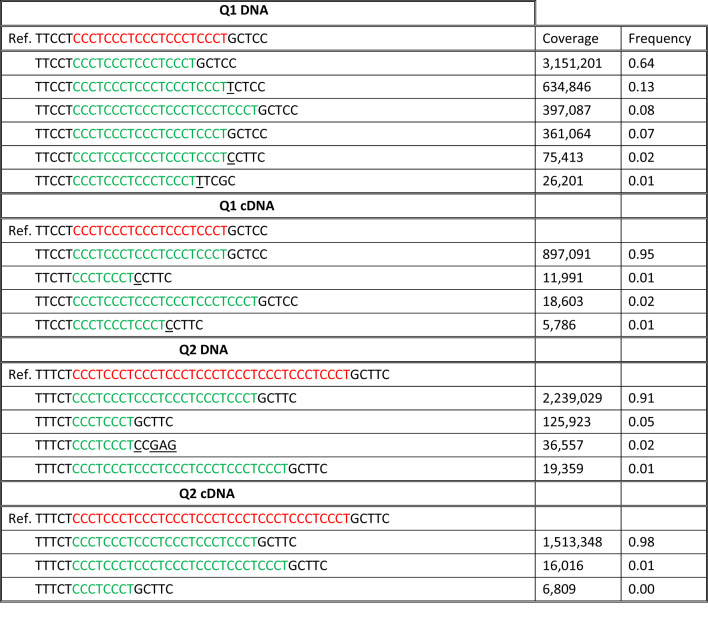
DNA and cDNA variants in the selected reads containing Q1 and Q2 regions with 5 bp long flanking sequences

The CCCT repeats in the selected reads and in the reference sequence (Ref.) are marked by green and red respectively. Underlined are bases which differ from ref on account of SNVs and other variants in the flanking sequences

These data show that the number of CCCT repeats varied considerably more in DNA than in cDNA (shown in Table 4). Among the indels deletions predominated, thus most DNA reads had reduced numbers of the repeats. The inserts appeared only in Q1 and more frequently in DNA (8%) than in cDNA (2%) with *p* < 0.001.

**Table 4:** Variability of the CCCT repeat numbers in Q1 and Q2 regions

		Frequency
Q1 DNA	Reference	0.20
	Indels	0.73
	Deletions	0.65
	Insertions	0.08
Q1 cDNA	Reference	0.95
	Indels	0.04
	Deletions	0.02
	Insertions	0.02
Q2 DNA	Reference	0
	Indels	0.98
	Deletions	0.98
	Insertions	0
Q2 cDNA	Reference	0
	Indels	0.99
	Deletions	0.99
	Insertions	0

The table shows the frequencies of the selected reads with repeat number corresponding to the reference sequence (Reference) and to indels. Only variants which appear in at least 2% reads are included

In Q2, all the reads contained reduced numbers of the repeats, which is probably the characteristic of the studied cell line [14]. But the length of indels differed in DNA and cDNA. To estimate this difference, we used the most frequent number of CCCT repeats (6) as a new reference (Ref#). We found that, in relation to Ref#, the indels appeared more frequently in DNA (7% deletions) than in cDNA (0% deletions) with *p* < 0.001 (Table 5). Thus, both Q1 and Q2 regions with reduced numbers of repeats were less likely to be expressed.

**Table 5:** Variability of the CCCT repeat number in the Q2 region with newly assumed reference (Ref#)

	Frequency
Total DNA	1
DNA Ref#	0.91
DNA indels#	0.07
DNA deletions#	0.07
DNA inserts#	0.01
Total cDNA	1
cDNA Ref#	0.97
cDNA indels#	0.01
cDNA deletions#	0
cDNA inserts#	0.01

(3) Variability in the flanking sequences

The flanking sequences (Fls) of the two CCCT repeat regions are very similar. In fact, most of the 10 upstream nucleotides and 6 downstream nucleotides related to Q1 and Q2 respectively are identical (underlined):

Q1: (5‘)TTTTCTTCCT (CCCT)_n_ GCTCCC(3‘).

Q2: (5‘)TGTTCTTTCT (CCCT)_n_ GCTTCC(3‘).

But upstream of the 10 marked base pairs and downstream of the 6 marked base pairs, the similarity between Q1 and Q2 abruptly vanishes. It seems that each microsatellite (CCCT)_n_, together with its 16 adjacent base pairs, constitutes one functional or potentially functional unit. Assembling the reads containing at least 10 nt upstream and 6 nt downstream Fls of the repeats, we found the frequencies of the Fls‘ variants (Table 6).

**Table 6:** Variability of the flanking sequences (including 10 nt upstream and 6 nt downstream) in the Q1 and Q2 regions. Relative numbers of the reads corresponding to the reference sequence (Reference) and differing from it (Variants)

	Q1 DNA	Q1 cDNA
Reference	0.79	0.97
Variants	0.15	0.02

	Q2 DNA	Q2 cDNA
Reference	1.00	1.00
Variants	0.02	0.00

Only 3‘ flanking sequences contained significant variants, most of which occurred at the position 41,878. In Q1 region, Fls in DNA were more variable (15%) than Fls in cDNA (2%) with *p* < 0.05. In Q2, SNVs appeared rarely and only in DNA. Thus DNA proved to be more variable than the corresponding cDNA in Fls as well as in the (CCCT)_n_ regions.

(4) Variability of the entire pRNA locus in the WI38 cells

To examine whether the sequence variants identified in this work were specific for the tumour-derived cells (HT1080) or represented a more common phenomenon, we examined the variability in WI38 cells, a normal diploid cell line derived from human fibroblasts. The results were also compared with the findings of our earlier work [14], in which we examined correlation of sequence variability with DNA methylation in the entire pRNA locus situated approximately between the position -2 kb and the transcription start site. Sequencing of the pRNA locus in WI38 revealed totally 23 single nucleotide substitutions and indels (against 32 for the same region in HT1080 cells), 13 of these variants appeared with frequency close to 100%. Among the latter, 8 were found also in the HT1080 cells (Table 7), including insert 41,006 and SNV_41,008_ belonging to 3’ end of Alu element. Remarkably, two SNVs common two both cell lines, 41,574 and 42,329, correlated with DNA methylation in HT1080 cells.

**Table 7:** Variants detected in the pRNA locus of WI38 cells

Position	Variant	Frequency	Coverage
**41,006**	**insG**	**1.00**	**299**
**41,008**	**G > C**	**1.00**	**299**
**41,208**	**C > T**	**0.62**	**627**
**41,214**	**T > C**	**0.42**	**615**
41,273	T > G	0.25	541
41,294	T > G	0.71	520
**41,310**	**delC**	**1.00**	**431**
**41,560**	**delT**	**1.00**	**244**
41,574*	**C > T**	**0.31**	**236**
**41,594**	**delT**	**0.97**	**233**
41,659	T > G	0.25	204
**41,663**	**insTCCTC**	**0.56**	**126**
**41,665**	**delTCCCTCC**	**0.10**	**174**
**42,255**	**C > G**	**1.00**	**679**
**42,256**	**G > C**	**1.00**	**674**
42,329*	**C > A**	**0.10**	**288**
42,398	insC	0.98	521
**42,400**	**C > G**	**0.98**	**505**
42,418	insGCG	0.99	534
42,451	insG	0.99	564
42,455	insCG	0.99	559
42,500	delA	0.99	562
42,553	delGCGGTC	0.34	435

Variants in bold were also discovered in HT1080. Asterisks indicate SNVs which correlated with DNA methylation in HT1080 (see Table 3)

Other variants, such as 42,329 fall near the putative miRNA binding site (hsa-miR-6724) (Table 6). Moreover, the number of CCCT repeats in the Q1 region proved to be highly variable owing to the insertions and deletions at positions 41,663 and 41,665 respectively. In the Q2 region, large deletions affecting tetrameric CCCT motifs were also observed. However, due to low coverage in this part of the URR in WI38, these events could not be confidently characterized and are not included in the table.

Thus we found that pRNA locus in the normal WI38 cells was less variable than in the transformed HT1080 cells, but > 50% single nucleotide substitutions and indels constituted in WI38 were shared with HT1080. Importantly, some of these shared variants (SNV_41,574_, SNV_42,329_, indels in the Q1 region) appeared in the putatively functional sequences.

## Discussion

The presented results indicate that deletions in (CCCT)_n_ regions and other variants within the human pRNA gene inhibit its expression. This may be occasioned by the effects of the observed variations on the pRNA transcription, as well as on its processing, for example, through altering the cleavage sites. Then our data would really reflect a reduction of expression for certain intermediary (or intermediaries) of pRNA produced from the DNA with deletions. Nevertheless, such reduction would also signify the diminishing level of the final product, the mature pRNA.

The results presented in the Tables 2, 3, 4, 5, 6 and 7 shed a new light on the data of our earlier study where we found a significant correlation between the variants in the pRNA encoding sequence and DNA methylation [14]. On the one hand, we can reaffirm our previous conclusion that sequence variability in the pRNA encoding region can alter the expression of the known inhibitor of ribosomal genes. For we show directly that the variability of the region situated upstream of the rDNA promoter has a significant impact on the expression of pRNA. According to our data, the SNV_41,574_, which converts CCC motif into CCT (Fig. 1; Table 2) and deletions which reduce the numbers of (CCCT) repeats in the Q1 and Q2 regions (Fig. 1; Tables 3, 4 and 5), being very frequent in the DNA, occur but rarely in the respective transcripts. Thus, both SNV_41,574_ and (CCCT) deletions inhibit pRNA expression, and apparently promote rDNA transcription.

On the other hand, we originally believed that DNA methylation in the entire Upstream Regulatory Region (URR) extended between the position -2 kb and the transcription start site, should inhibit transcription of ribosomal genes [14]. But, there was no strong evidence for that. Only certain methylated CpG islands within the rDNA promoter (e.g., at the position -133 in mouse cells) have been experimentally identified as specific targets of the regulatory methyltransferases [23]. The data of the present study indicate that the variants located within the URR (Fig. 1C, D), which negatively correlated with DNA methylation (Table 2), also positively correlate with the transcription of pRNA and, therefore, must facilitate the 47S rDNA transcription. In our earlier work [14], we also suggested that the G-quadruplexes in the intact Q1 and Q2 regions should inhibit production of the pRNA transcript, and that various deletions of the CCCT repeats should destabilize the quadruplexes and promote pRNA expression. Now we find that such deletions, on the contrary, considerably diminish expression of pRNA (Tables 4 and 5).

To make sense of our earlier and present results, we will consider the following facts. Firstly, it is known that the length of short tandem repeats in various transcription regulating regions is a significant factor in the promoter activity [24–26]. Secondly, it was found in experiments with push-apart constructs that changing the distance between the TSS of the 47S rDNA region and the proximal terminator T_0_ by as little as 4 nucleotides can switch the rDNA promoter between active and silent states [27]. Thirdly, the (CCCT)_n_ regions, which consist of the regular stretches of pyrimidine repeats, are optimal for the formation of parallel and antiparallel RNA-DNA:DNA triplexes. Such structures composed from the (CCCT)_n_ /(GGGA)_n_ regions of DNA and the (GGGA)_n_ sequences of pre-pRNA, like the triplex formed between T_0_ and the complementary sequence of the mature pRNA, may transiently anchor the primary transcript to facilitate its processing and to prevent its drifting away to other rDNA units. Accordingly, we suppose that the tetrameric deletions in the Q1 and Q2 regions get suppressed, for they would damage the pRNA function by reducing the probability of the triplex formation. Signal to this suppression may be provided by shortening the distance, e.g., between SPr and T_0_. It must be mentioned that the pRNA transcription would hardly be prevented by the construction of triplex, since its RNA component is rather short-lived: according to our earlier study, the half-life time of various parts of pre-pRNA is about 5 min [28].

Although the role of triplex formation based on Q1 and Q2 regions remains hypothetical, the data of this and our earlier publications, show that the transcription of pRNA, as well as rDNA, significantly depends on the variability of the pRNA encoding sequence situated 1–2 kb upstream of the rDNA promoter. Additionally, our data indicate that DNA methylation in these upstream loci can reduce the transcription of pRNA and thus promote the expression of rDNA. The presence of certain variations in the DNA sequence seems to be required for normal regulation of the rDNA synthesis.

## Conclusions

Our study demonstrates that sequence variability of the human pRNA gene significantly impacts its expression. We show that specific variants, such as SNV41,574 and deletions in Q1 and Q2 regions of short tandem repeats (CCCT)_n_, are frequent in DNA but rarely detected in transcripts, suggesting selective inhibition of pRNA expression and, consequently, promotion of rDNA activity. Furthermore, our findings revise our previous supposition about the role of G-quadruplex structures and DNA methylation at the loci situated upstream of the promoter. Thus our new findings indicate that methylation at the position 41,574 should promote the 47S rDNA transcription. This work emphasizes the regulatory importance of the pRNA encoding region, located 1–2 kb upstream of the rDNA transcription start site.

## Data Availability

No datasets were generated or analysed during the current study.

## Abbreviations

CPE: Core promoter element
Fls: Flanking sequences
IGS: Intergenic spacer
mpRNA: Mature promoter RNA
NoRC: Nucleolar remodelling complex
pRNA: NoRC-associated RNA or promoter RNA
rDNA: Ribosomal DNA
pol I: RNA polymerase I
SNV: Single nucleotide variant
SPr: Spacer promoter
TIP5: TTF-I-interacting protein 5
TSS: Transcription start site
TTFI: Transcription termination factor I
UCE: Upstream control element
